# Hypothalamic Regulation of Obesity

**DOI:** 10.3390/ijms222413459

**Published:** 2021-12-15

**Authors:** Rosalía Rodríguez-Rodríguez, Cristina Miralpeix

**Affiliations:** 1 Basic Sciences Department, Faculty of Medicine and Health Sciences, Universitat Internacional de Catalunya, 08195 Sant Cugat del Vallès, Spain; 2 INSERM, Neurocentre Magendie, U1215, University of Bordeaux, 3300 Bordeaux, France; cristina.miralpeix@inserm.fr

Obesity has now reached pandemic proportions and represents a major socioeconomic and health problem in our societies. Up to now, obesity has been considered a medical issue only in high-income countries, but this disease is now dramatically on the rise in low- and middle-income countries, particularly in urban settings, affecting both adults and the pediatric population [1]. 

The simplest definition of overweight and obesity is an excessive accumulation of fat due to an imbalance between the energy in and the energy out, and this adiposity represents a risk to health [1]. But the whole picture is more complex than this, and obesity is a marker of a serious metabolic dysregulation that involves several diseases such as type 2 diabetes, cancer, cardiovascular pathologies, and most recently for COVID-19 infection [1,2]. Since the current approaches to combatting obesity and its complications have limited clinical effectiveness, gaining insight into the cellular and molecular basis of obesity could lay the foundations for the development of new strategies to prevent metabolic disruption and to treat this somewhat unaddressed medical issue. 

In the last few decades, it has been strongly demonstrated that the hypothalamus is the master regulator of energy homeostasis. The hypothalamus contains hormone- and nutrient-sensing nuclei that organize central and peripheral responses for maintaining normal body weight, food intake, energy expenditure, and nutrient partitioning. Within the hypothalamus, specialized sub-populations of neurons are connected to each other and to various extrahypothalamic regions to coordinate energy balance. Evidence has also highlighted the participation of non-neuronal populations (i.e., microglia and astrocytes), and even the interesting cross-talk between these types of brain cells, whose disruption leads to insulin resistance and obesity. In this Special Issue, we report on the most recent insights into the hypothalamic circuitries and pathways involving neurons [3,4,5,6,7], astrocytes [8,9], and microglia [10,11] in obesity development and associated complications. The emerging contribution of astrocyte–neuron [9] and microglia–neuron cross-talks [12] in the hypothalamus and the contribution of microbiota and the gut-brain axis controlling food intake and energy homeostasis [13] are also presented in this Special Issue (Figure 1).

Regarding the importance of hypothalamic targets in obesity development, Quiñones et al. [3] elegantly review the most relevant studies focusing on the deacetylases sirtuins in hypothalamic neurons, SIRT1 and SIRT6, as multifaceted mediators of energy metabolism, controlling processes such as food intake, food preference, puberty, body weight, adiposity, and glucose and insulin homeostasis. Importantly, the complexity of the hypothalamic nuclei in obesity development is supported by the fact that the effect of SIRT1 on energy balance depends on the neuronal type where it is acting. For instance, lacking SIRT1 or SIRT6 in POMC neurons affects energy expenditure and adiposity; however, SIRT1 in AgRP neurons specifically affects eating behavior, and in oxytocin neurons in the PVN controls diet preference. In addition to sirtuins, Fosch et al. [4] provide new insights into other targets in SF1 neurons of the ventromedial hypothalamus that play a critical role not only in body weight control and adiposity, but also in glucose tolerance and insulin and leptin sensitivity, with minimal effects on food intake and differential actions between male and female mice. The authors also report how nutrient sensors such as AMPK and SIRT1, glutamatergic transmission, synaptic receptors, and mediators of autophagy in SF1 neurons are promising therapeutic targets against obesity and diabetes, reinforcing the “central role” of the hypothalamus in the control of peripheral metabolism beyond the regulation of feeding behavior [4]. In this sense, Fukumura et al. [5] found new evidence in this special issue about the effects of a novel hypothalamic small protein, named neurosecretory protein GL (NPGL), on glucose and insulin homeostasis. In this study, overexpression of *Npgl* in the mediobasal hypothalamus of mice improved glucose tolerance and attenuated insulin resistance and hyperglycemia under high-fat diet (HFD) exposure, without significant changes in food intake. The concomitant increased mRNA expression levels of galanin (a neuropeptide that co-localizes with NPGL in arcuate neurons and regulates glucose homeostasis) suggests that both peptides could regulate each other for these actions [5].

When investigating the hypothalamic regulation of obesity, in addition to the identification of potential targets mediating the homeostatic circuits, it is also important to explore the hedonic circuits mediating energy balance, identifying the salient neural, hormonal, and humoral components involved, as remarkably reviewed by Gastelum et al. [7]. They explore the sexual dimorphism on these circuits, paying special attention to the role of two emerging neuropeptides, the nociception/orphanin FQ and the pituitary adenylate cyclase-activating polypeptide (PACAP) in the neuronal activity regulation in positive and negative energy states. Jang et al. [6] also investigate leptin-mediated feeding circuits in hypothalamic neurons, identifying the expression of the angiopoietin-like growth factor (AGF) in POMC neurons of the arcuate nucleus as a downstream factor involved in leptin signaling in the hypothalamus. 

Hypothalamic circuits controlling energy balance in response to feeding are also mediated by the gut-to-brain nutrient signaling, whose disruption leads to obesity. As extensively reported by Romani-Perez et al. [13], aberrant feeding patterns or unhealthy diets might alter gut microbiota–diet interactions and modify nutrient-sensing information from the gut to the hypothalamus, impairing energy homeostasis. They identify microbiome-based strategies to improve the gut-brain axis function and hence combat obesity. 

An already well-established concept in the neurobiology of obesity is that neurons are not the only characters playing a main role in the response processing and the transmission of information. This is also true in the hypothalamus, where a maladaptive interaction between neurons and their surrounding microglia and astrocytes likely contribute to the development and progression of obesity [9,12]. The perspective elegantly presented by Leon et al. [12] discusses recent evidence that sheds light on the cellular and molecular mechanisms underlying microglia–neuron communication in hypothalamic circuits that are crucial for body weight and food-intake control in response to HFD exposure. Interestingly, microglia, as key players in the immunological response, are sensitive to fuel substrate and can influence neuronal activity in part via cytokines. However, the maladaptive microglia–neuron cross-talk observed in response to overnutrition seems to involve synergy with astrocytes, which are key components of the tripartite synapse, as discussed below. In addition to these cellular interactions, Mendes et al. [10] examine the subtypes of microglia that may be involved in HFD-induced hypothalamic inflammation in the early stages of the disease. They also remarkably discuss which models can be useful for obtaining the most reliable data when exploring distinct subsets of microglia, and clarify the changes in hypothalamic microglia signature in response to HFD-induced obesity based on novel transcriptomic analysis. The importance of microglia activation in the hypothalamus in energy balance has also been reported by Lopez-Gambero et al. [11]. They found that glial activation and metabolic dysfunction in the hypothalamus of a mouse model of Alzheimer’s disease (5XFAD mouse) leads a negative energy balance and lower insulin and related hormones in plasma, contributing to the development of the pathology. Interestingly, this phenotype was more marked in female than male mice, indicating useful information for early detection of the disease and sexual dimorphism in the pathophysiology of Alzheimer’s disease. 

The function of hypothalamic astrocytes and the interplay between astrocytes and neurons are also becoming very important in the study of obesity development. In this special issue, Song et al. [8] show the ability of the adipose tissue-derived hormone adiponectin to control nutrient metabolism in hypothalamic astrocytes by enhancing glucose uptake, glycolytic activity, and lactate and ketone body production. In this study, central administration of adiponectin increases the number of astrocytes in the hypothalamus of mice. The most relevant findings regarding molecular mechanisms by which hypothalamic astrocytes are involved in the pathogenesis of obesity have been well reviewed by Gonzalez-Garcia et al. [9]. They expose the idea of how a disruption in the astrocyte–neuron communication within the hypothalamus is altered by exposure to obesogenic insults and the impact this has on obesity development. In addition, they point out the emerging idea that astrocytes are heterogeneous and that there might exist subpopulations that contribute to specific actions. Therefore, the identification of specific hypothalamic astrocyte subpopulations might be critical for the pathophysiology of obesity [9]. The study of the potential role of other brain cells and how they are interconnected with glia and neuronal cells in the hypothalamic regulation of obesity is also promising (e.g., tanycytes and endothelial cells) [12]. 

Therefore, the manuscripts published in this Special Issue provide new insights into the hypothalamic neuronal and non-neuronal pathways involved in the pathophysiology and development of obesity and related diseases such as diabetes (Figure 1). Via four original papers, six review manuscripts, and one perspective, this Special Issue will substantially contribute to the identification of novel therapeutic targets in the hypothalamus and novel approaches to combatting the progression of human obesity. 

## Figures and Tables

**Figure 1:**
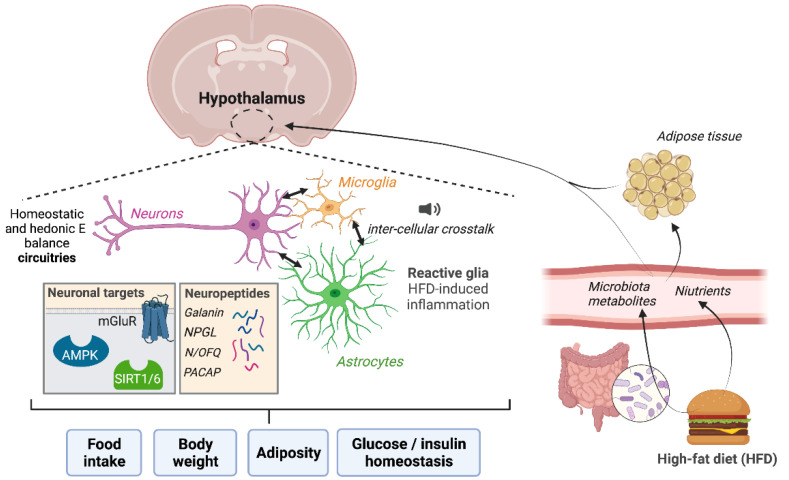
In this Special Issue, we report on the most recent insights into the neuronal targets and neuropeptides of the hypothalamic circuitries involved in obesity development and progression. In addition, the emerging contribution of astrocyte-neuron and microglia-neuron cross-talks in the hypothalamus in response to a high-fat diet, and the implication of the gut-brain axis controlling food intake and energy homeostasis are also presented
